# Measuring patient outcomes in chronic heart failure: psychometric properties of the Care-Related Quality of Life survey for Chronic Heart Failure (CaReQoL CHF)

**DOI:** 10.1186/s12913-017-2452-4

**Published:** 2017-08-07

**Authors:** Paul van Kessel, Dolf de Boer, Michelle Hendriks, Anne Marie Plass

**Affiliations:** 1 Netherlands Institute for Health Services Research (NIVEL), Utrecht, Netherlands; 2 University Medical Center Göttingen (UMG)/ Georg-August-University, Institute of Medical Psychology and Medical Sociology, Göttingen, Germany

## Abstract

**Background:**

The Care-Related Quality of Life survey for Chronic Heart Failure (CaReQoL CHF) is a newly developed patient-reported outcome measure (PROM) that measures care-related quality of life in patients diagnosed with chronic heart failure. This study describes the psychometric properties of the questionnaire and its relationship with disease severity and global rating of quality of care.

**Method:**

Insurance companies selected patients with a recorded diagnosis of chronic heart failure and for whom the hospital submitted a billing statement in the last year. Exploratory factor analysis, Cronbach’s alpha and item-rest correlation were used to construct the CaReQoL CHF. Construct validity was assessed by examining the mean values of the CaReQoL CHF scales for the categories of the widely-used New York Heart Association (NYHA) functional classification and by correlating the global rating of quality of care with the CaReQoL CHF scales.

**Results:**

One thousand eighteen patients with chronic heart failure filled out the CaReQoL CHF (RR: 35.7%). The CaReQoL CHF consists of 20 items and three scales: social and emotional problems, physical limitations, and being in safe hands. The mean scores of the three scales differed significantly for the NYHA categories, particularly for the social-emotional problems and physical limitation scales. The ‘being in safe hands’ scale showed a moderate positive correlation with the global rating of quality of care.

**Conclusions:**

The CaReQoL CHF is a concise and valid PROM that matches patients’ priorities in healthcare. It adds a new element to existing quality of life questionnaires for patients with chronic heart failure, that is ‘being in safe hands’ scale. This scale is relevant for patients because they experience anxiety and tension about their condition. Future research should determine whether the CaReQoL CHF can help healthcare providers in daily practice to focus treatment on outcomes of care that are relevant to individual patients.

## Background

Healthcare providers have to take patients’ preferences and needs into account [1]. Accordingly, patient surveys are used to measure care outcomes as perceived by patients [2]. These surveys are called patient-reported outcome measures (PROMs).

An advantage of using PROMs in clinical practice is that the outcomes can help healthcare providers monitor individual patients in between consultations and evaluate and manage their treatment [3, 4]. In addition, group-level data of PROMs for different treatments may help patients and doctors choose between treatments [5]. Finally, PROMs are considered to provide an important method of measuring quality of care by comparing average PROM scores between providers [6].

In people with a chronic illness, measurements of physical, mental and/or social functioning and/or (health-related) quality of life are often used to monitor outcomes of care [7, 8]. Widely used generic quality of life measures such as SF-36 and EQ-5D [9, 10] are considered too generic to capture aspects of care that matter to chronic patients [11, 12]. Disease-specific quality of life measures on the other hand, are not always developed with patient involvement [13]. This can lead to inappropriate outcome selection and can result in unimportant or misleading information and wasted resources [14]. It is widely acknowledged that it is pivotal to involve patients in the selection of outcomes and the development and validation of patient surveys such as PROMs, because this helps to capture the full spectrum of the patient perspective [11, 14–17].

One such group with chronic disease is people with chronic heart failure. Patients with chronic heart failure suffer from shortness of breath, fatigue and swelling in the ankles and legs [18]. In the Netherlands, approximately 1% of the population (roughly 130,000 people) suffer from chronic heart failure, mostly over the age of 75 [19]. The lifetime prevalence is about 20-30% [19].

Kelkar et al. [20] identified 31 PROMs for chronic heart failure and reported several relevant measurement properties such as reliability, validity, responsiveness, diversity in performance, feasibility, interpretability and prognostic value of these questionnaires. The Minnesota Living with Heart Failure Questionnaire (MLHQ; [21]) and the Kansas City Cardiomyopathy Questionnaire (KCCQ; [22]) met these criteria best. These questionnaires have been developed and assessed in the United States. However, it is possible that differences exist between countries in the extent to which a PROM addresses the relevant and important aspects for patients, a phenomenon known as cross-cultural validity. Therefore, before implementing an existing PROM, it is important to evaluate the PROM and determine whether it captures the patient perspective in the designated country. If not, an existing PROM may be modified or a new PROM may be developed.

In the present paper we report on the development of a new PROM for chronic heart failure, which was deemed necessary as Dutch patients reported a key priority that was not captured in existing PROMs. This key priority was whether patients felt they were “safe”, that is, properly monitored such that they felt comfortable and confident to proceed with daily activities.

Based on focus groups with heart failure patients and inspired by the MLHQ and the KCCQ, we developed the Care-Related Quality of Life survey for Chronic Heart Failure (CaReQoL CHF). In the present paper, we examine the psychometric properties and construct validity of the CaReQoL CHF. Our research questions were:

1. Psychometric properties: what are the psychometric properties of the CaReQoL CHF in terms of dimensional structure and Cronbach’s alpha?
2. Validity: how are the reported outcomes measured by the CaReQoL CHF related to disease severity and global rating of quality of care?

## Method

### Development of the CaReQoL CHF

In the literature review, we found that the following themes were common in existing disease-specific PROMs for chronic heart failure: physical limitations, participation in society, and feelings of anxiety or tension [23–26]. We asked Dutch patients with heart failure what are important health outcomes for them in order to establish cross-cultural validity. We confirmed the common themes in two focus group discussions with a total of nine patients. Interestingly, ‘being in safe hands’ emerged as an additional theme that was not covered in the existing PROMs. Patients indicated that they were reminded of their condition on a regular basis and that they often feel anxious or tense given that their heart problems could deteriorate at any moment. Healthcare providers were able to reduce feelings of anxiety or tension by watching over the patients. For example, by telemonitoring constantly how the heart functions and by proactively contacting the patient when needed. As such, we decided to develop a new PROM to address these issues. Cognitive interviews were held to enhance the reliability and validity and to verify that the questions were concise, simple, and interpreted as intended [27–30]. Detailed results of the focus group discussions and cognitive interviews have been reported elsewhere in Dutch [31]. The initial CaReQoL CHF (before item reduction) consisted of 34 items on a five-point scale (never, seldom, sometimes, often, always and not applicable). The questions in this version focus on experiences of patients in the last 4 weeks.

### Data collection

We selected 31 hospitals with a large number of declarations for heart failure. In each of these hospitals, insurance companies randomly selected roughly 100 patients with a recorded diagnosis of chronic heart failure and for whom the hospital submitted a billing statement in the last year (these were either outpatients or patients admitted to the hospital). We selected patients from several hospitals via insurance companies because we wanted to get more insight in possible differences between hospitals and, more pragmatically, insurance companies were willing to help us get access to patients. Patients were able to choose whether they wanted to fill in the questionnaire online or on paper (mixed-mode). In week 1 patients were sent an invitation to fill in the online version of the CaReQoL CHF. In week 2 patients were sent a thank you/reminder note. In the event of non-response, patients were sent a paper version of the CaReQoL CHF in week 5 and a final reminder in week 7. No ethical approval and consent for publication for the study was necessary, as research by a non-encroaching survey such as used in this study is not subject to the Dutch Medical Research Involving Human Subjects Act (WMO).

### Analyses

In the present paper, the reliability and validity of the CaRe-QoL CHF will be addressed. Furthermore, the dimensional structure was assessed with exploratory factor analysis (promax rotation), including Bartlett’s test of sphericity and Kaiser-Meyer-Olkin as a measure of sampling adequacy. We used listwise deletion for the factor analysis, meaning that only respondents with no missing values were taken in account. This avoids imputation of missing items. As a rule of thumb, items with a factor loading higher than .6 were included in the CaReQoL CHF. Cronbach’s alpha and item-rest correlation gave insights into the test reliability of the scales and redundant items [32]. The self-reported New York Heart Association (NYHA) classification was used as a reference for assessing (criterion) validity by examining the means of the CaReQoL CHF scales for different NYHA categories using ANOVA. The NYHA classification is a measure of the severity of a patient’s heart failure in terms of dyspnoea and fatigue [33]. Respondents assigned themselves to one of four categories, ranging from no limitation in physical activity to severe limitations (see Table 1). A second aspect of criterion validity was the associations between the CaRe-QoL CHF and the global rating of quality of care. Patients were asked ‘How would you rate the healthcare you received in this hospital for chronic heart failure? A 0 means very bad and a 10 means excellent’ [34, 35]. We examined the associations with the scores on the CaRe-QoL CHF using Pearson’s correlations, which were also used to assess association between the CaReQoL CHF scales.

**Table 1:** Sample description in terms of age, sex and categories of the New York Heart Association functional classification

Age m (sd)	71.7 (11.8)
Sex n (%)	
Male	585 (59.3%)
NYHA n(%)	
1 - No limitations due to dyspnoea or fatigue	224 (23.3%)
2 - Dyspnoea or fatigue with normal physical activity	299 (31.2%)
3 - Dyspnoea or fatigue with slight physical activity	357 (37.2%)
4 - Dyspnoea or fatigue with every physical activity and in rest	80 (8.3%)

## Results

### Research question 1: what are the psychometric properties of the CaReQoL CHF?

The CaReQoL CHF was filled out by 1018 respondents (RR: 35.7%). Table 1 describes the sample in terms of age, gender and NYHA classification. Bartlett’s test of sphericity was significant (*p* < .001), indicating that the variables correlated highly enough for factor analysis. The Kaiser-Meyer-Olkin value was high, with a score of .93, indicating factor analysis should yield distinct and reliable factors. Following factor analysis (*N* = 249, listwise deletion) and reliability analysis, 20 items were retained in the final version of the CaReQoL CHF. The results of the factor- and reliability analyses for those items are presented in Table 2.

**Table 2:** Factor and reliability analysis of items in the final version of the CaReQoL CHF

Item	Factor loadings and item-rest correlation (if included in scale)					
	Social and emotional problems		Physical limitations		Being in safe hands	
	Factor loading (*N* = 249)	Item-rest correlation (summary α for scale = 0.93, *N* = 896)	Factor loading (*N* = 249)	Item-rest correlation (summary α for scale = 0.92, *N* = 935)	Factor loading (*N* = 249)	Item-rest correlation (summary α for scale = 0.74, *N* = 955)
Because of my heart condition…						
I do everything slower than I would like			0.86	0.71		
I don’t have much energy			0.82	0.72		
spontaneous activities are difficult			0.71	0.69		
During the past 2 weeks…						
I was worried something might happen to my heart	0.78	0.69				
I have slept poorly because of my heart condition	0.70	0.67				
the hospital care made me feel safe					0.62	0.42
I had the feeling that every day could be my last	0.73	0.68				
During the past 2 weeks, my heart condition has meant…						
I had difficulty walking			0.85	0.77		
I had difficulty cycling			0.83	0.72		
I had difficulty with the housekeeping			0.77	0.78		
I had difficulty enjoying my family life	0.70	0.76				
I had difficulty in my contact with family or friends	0.59	0.71				
I had difficulty enjoying life	0.74	0.77				
During the past 2 weeks, my heart condition has made me feel…						
miserable	0.82	0.79				
incapable of doing things	0.73	0.80				
irritable	0.70	0.69				
anxious	0.98	0.79				
My care providers keep a close eye on me					0.72	0.49
I trust that medical help will arrive on time when needed					0.79	0.59
I am confident about the healthcare I receive					0.80	0.62

Factor analyses resulted in three scales: (1) social and emotional problems, (2) physical limitations and (3) being in safe hands. One item about having contact with friends and family was retained despite the slightly lower factor loading (.59), because patients talked about the subject a lot in the focus group discussions. Table 3 shows which items were not included in the CaReQoL CHF and for what reason.

**Table 3:** Factor analysis of items excluded from the CaReQoL CHF

Item	Factor loadings		
	Social and emotional problems	Physical limitations	Being in safe hands
I’m enjoying my life	−0.53		
I can live my life the way I want to		−0.55	
Because of my heart condition…			
I am anxious^a^	0.90		
my future feels uncertain	0.59		
coping with friends and family is too stressful	0.40		
I appreciate life more			0.39
I can’t eat what I want	0.27		
I am a burden for others	0.34		
I had difficulty doing paid work^b^			
I had difficulty doing voluntary work^b^			
During the past 2 weeks…			
I experienced shortness of breath		0.51	
I was able to do the things I want		−0.44	
During the past 2 weeks, my heart condition has made me feel…			
I had difficulty concentrating	0.46		
I had difficulty getting out of the house	0.52		
that others were being over-protective		0.33	
My heart condition prevents me from…			
living a carefree life		0.36	

^a^not included due to inclusion of a similar item

^b^deleted due to too many missing values

Correlation between the scales ranged from −.08 to .63 (*p*-values <.05). Social and emotional problems were positively related to physical limitations (*r* = .63) and negatively related to ‘being in safe hands’ (*r* = −.24). Physical limitations were negatively associated with being in safe hands (*r* = −.08).

### Research question 2: relationship with disease severity and patient satisfaction

**Fig. 1:**
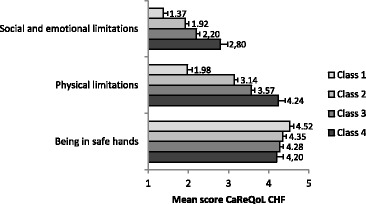
Mean scores (and 95% confidence interval) on the CaReQoL CHF scales for different NYHA classification categories. Social and emotional limitations, F=74.64, df=847, η^2^=0.21, *p*<0.001, Physical limitations, F=214.27, df=878, η^2^=0.42, *p*<.001, Being in safe hands, F=5.77, df=900, η^2^=0.02, *p*<0.001

There were significant differences (*p* < .05) for every scale of the CaReQoL CHF between the four NYHA categories (Fig. 1). Patients in higher classification scales (meaning more symptoms) had higher scores on the social and emotional limitation and physical limitation scales. The (significant) differences for the ‘being in safe hands’ scale on the NYHA classification were not large, suggesting that this scale is largely independent of the severity of the disease.

### Correlation with patient satisfaction

The scales social and emotional problems and physical limitations showed a small negative association with global rating of quality of care (*r* = −.16 and −.10 respectively, *p*-values < .05). This means that the more social and emotional problems and physical limitations a patient experiences, the lower their global rating of quality of care. Being in safe hands was positively associated with global rating of care (*r* = .43, *p*-value < .05). Therefore, the more they felt they were in safe hands, the higher the global rating of quality of care.

## Discussion

In the present study we developed a PROM that measures care-related quality of life in patients diagnosed with chronic heart failure. The study resulted in a concise (20-item; see Table 2) and valid questionnaire that matches patients’ priorities in healthcare, the Care-Related Quality of Life survey for Chronic Heart Failure (CaReQoL CHF). Twenty items is shorter than most other questionnaires and the content of the CaReQoL CHF is comparable with the MHLF and KCCQ. The CaReQoL CHF consists of three scales: social and emotional problems, physical limitations and being in safe hands. In this study, we established the reliability and construct validity of the CaReQoL CHF. Cronbach’s alphas of the scales were sufficiently high [32]. The scales for social and emotional problems and for physical limitations were positively associated with severity of the disease, whereas the ‘being in safe hands’ scale was moderately and positively associated with global rating of quality of care. The combination of these associations suggest that the CaReQoL CHF is a valid instrument for measuring relevant outcomes of care from a patient’s perspective. The CaReQoL CHF provides the field of heart failure with a promising instrument to evaluate perceived outcomes of healthcare in this patient group.

The scale ‘being in safe hands’ is an element not measured with existing quality of life questionnaires for patients with chronic heart failure. Being in safe hands was mentioned as an important outcome in focus groups with Dutch patients with heart failure [31]. This raises the question whether being in safe hands is something typical Dutch or if this aspect is relevant to patients in other countries too. Regardless of the severity of their symptoms, patients should be able to feel they are safe – and in safe hands - thanks to the healthcare provided. This is particularly relevant because heart failure is a progressive disease that cannot be cured.

Anxiety and tension in patients (e.g. in anticipation of sudden events such as a myocardial infarction) can be alleviated by knowing that healthcare providers are watching over them. This is also found in a meta-analysis [36] and a study by Renzi et al. ([37], pp. 716-717) which stated that a good doctor-patient relationship can improve healthcare outcomes and the patients’ health-related quality of life. For heart failure, the health-related quality of life can be improved by monitoring patients at home, using the CaReQoL CHF between consultations.

We regarded ‘being in safe hands’ as an outcome of care. However, it could also be argued that it is a process aspect of care because it comprises aspects of care delivery and care coordination [38]. In addition, being in safe hands falls outside the range of domains of health-related quality of life that are common to existing questionnaires (which are social, emotional and physical domains) [39]. Associated with the global rating of care, the ‘being in safe hands’ scale is an interesting new aspect in the area where the process and the outcome of care intersect.

A limitation of this study was that the (self-reported) NYHA classification was used for criterion validation, although we concede of course that there are no real gold standards in the validation of outcome measurements [40, 41]. Furthermore, this study was performed in a cohort of patients receiving hospital care, whilst some patients with chronic heart failure (in the Netherlands) only receive care from their general practitioner. This latter group probably includes patients with less severe complaints and therefore their answers on the CaReQoL CHF might differ from the answers of patients receiving hospital care. However, given that our population comprises all of the four NYHA categories, we expect no significant differences for instrument validation purposes.

This study is to be considered a starting point in the development of the CaReQoL CHF. It would be valuable to perform confirmatory factor analysis and to assess the convergent validity of the CaReQoL CHF by comparing scores with those on questionnaires such as the MLHQ and KCCQ. Future research may also employ longitudinal measurement with the CaReQoL CHF to assess the responsiveness of the CaReQoL CHF. Responsiveness addresses the ability of a PROM to detect change over time in the construct measured [42]. The present study is based on a single measurement in order to validate the CaReQoL CHF. More measurement points over time with the same patients would provide insight into the way the CaReQoL CHF responds to the effects of a treatment and whether these effects differ between patient groups, treatment options or healthcare providers. Finally, it would be relevant to determine whether the CaReQoL CHF can be used to monitor and adjust treatments, and whether incorporating results of the CaReQoL CHF into clinical daily practice would help improve the quality of care.

## Conclusion

We developed a new instrument for measuring care-related outcomes in patients suffering from chronic heart failure, the CaReQoL CHF. It is a concise and valid PROM that matches patients’ priorities in healthcare. The instrument requires further validation, but possesses at least one promising feature that may be relevant for chronic heart failure patients in general, which is that it covers the theme ‘feeling in safe hands’. This theme was identified as being very important by the patients, but lacking in existing PROMs. In addition, the instrument is rather short compared to other existing instruments which is generally considered desirable. Future research should further establish the validity and reliability of the instrument by comparing scores with those on other questionnaires and possibly confirmatory factor analysis.

## Abbreviations

CaReQoL CHF: Care-Related Quality of Life survey for Chronic Heart Failure
NYHA: New York Heart Association
PROM: Patient-Reported Outcome Measure
